# Brain Network Alterations in Chronic Spinal Cord Injury: Multilayer Community Detection Approach

**DOI:** 10.1089/neur.2024.0098

**Published:** 2024-11-06

**Authors:** Farzad V. Farahani, Lukman E. Ismaila, Cristina L. Sadowsky, Haris I. Sair, Li Min Chen, Visar Belegu, James J. Pekar, Martin A. Lindquist, Ann S. Choe

**Affiliations:** ^1^ Department of Biostatistics, Johns Hopkins Bloomberg School of Public Health, Baltimore, Maryland, USA.; ^2^ Russell H. Morgan Department of Radiology and Radiological Science, Johns Hopkins School of Medicine, Baltimore, Maryland, USA.; ^3^ F.M. Kirby Research Center for Functional Brain Imaging at Kennedy Krieger Institute, Baltimore, Maryland, USA.; ^4^ International Center for Spinal Cord Injury at Kennedy Krieger Institute, Baltimore, Maryland, USA.; ^5^ Department of Physical Medicine and Rehabilitation, Johns Hopkins School of Medicine, Baltimore, Maryland, USA.; ^6^ The Malone Center for Engineering in Healthcare, The Whiting School of Engineering, Johns Hopkins University, Baltimore, Maryland, USA.; ^7^ Vanderbilt University Institute of Imaging Science, Vanderbilt University, Nashville, Tennessee, USA.; ^8^ Department of Radiology and Radiological Sciences, Vanderbilt University Medical Center, Nashville, Tennessee, USA.

**Keywords:** cortical reorganization, functional connectivity, graph theory, mesoscale, rs-fMRI, spinal cord injury

## Abstract

Neurological recovery in individuals with spinal cord injury (SCI) is multifaceted, involving mechanisms such as remyelination and perilesional spinal neuroplasticity, with cortical reorganization being one contributing factor. Cortical reorganization, in particular, can be evaluated through network (graph) analysis of interregional functional connectivity. This study aimed to investigate cortical reorganization patterns in persons with chronic SCI using a multilayer community detection approach on resting-state functional MRI data. Thirty-eight participants with chronic cervical or thoracic SCI and 32 matched healthy controls were examined. Significant alterations in brain community structures were observed in the SCI cohort, particularly within the sensorimotor network (SMN). Importantly, this revealed a pattern of segregation within the SMN, aligning with borders of representations of the upper and lower body and orofacial regions. The SCI cohort showed reduced recruitment and integration coefficients across multiple brain networks, indicating impaired internetwork communication that may underlie sensory and motor deficits in persons with SCI. These findings highlight the impact of SCI on brain connectivity and suggest potential compensatory mechanisms.

## Introduction

Neurological recovery from spinal cord injury (SCI) depends on the injury extent and degree of spinal cord preservation. Partial preservation is crucial, as complete recovery is unlikely without it.^1,2^ However, recovery, especially in locomotion, is not directly proportional to the amount preserved.^2–4^ This suggests that other factors, such as cortical reorganization, play a significant role, highlighting the need to understand these mechanisms better.

SCI inevitably affects the brain through direct neurological disruptions and secondary cortical reorganization.^5^ A common pattern is the expansion of spared limb representation into deprived cortical regions. For instance, lateral hemisectional cervical lesions in rats enhance unimpaired forepaw representation in adjacent regions.^6^ Similar patterns are also observed in humans.^7–11^ These observations are enabled by advanced imaging techniques like resting-state functional MRI (rs-fMRI).^12^

Rs-fMRI measures brain activity while a person is at rest, detecting spontaneous and organized activity through changes in blood flow and oxygenation.^13^ Hence, it is particularly useful for investigating cortical reorganization in persons with SCI, as it provides consistent imaging across diverse cohorts with varying degrees of residual motor and sensory functions.^14–16^ Studies suggest that rs-fMRI can detect changes in functional connectivity as early as one week into an intervention,^17–19^ making it a promising tool for monitoring and understanding brain network changes in SCI.

Graph theory analysis offers a quantitative approach to assessing changes in brain network organization in SCI.^20,21^ Brain networks are represented as graphs, with brain regions as nodes and their connections as edges. Research consistently shows that SCI leads to brain network reorganization, impacting both functional and structural connectivity.^14,22–25^ However, discrepancies exist, with some reporting increased global efficiency and decreased local efficiency, while others observe reductions in both.^22,24^ These differences may arise from varying study populations and connectivity types studied, requiring further investigation.

In this exploratory study, we build on graph theory by applying the multilayer community detection algorithm to analyze SCI data.^21^ This method incorporates both intra- and interlayer interactions, providing a more detailed view of brain network organization. It is particularly effective at detecting subcommunities that single-layer analyses may miss. Previous research has demonstrated its ability to capture functional connectivity changes in both healthy and diseased brains.^26–29^ By treating each subject as a separate layer, the algorithm enables cross-individual analysis, making it well-suited for identifying patterns linked to traits or conditions like cortical reorganization in SCI. To our knowledge, this is the first study to apply this approach to SCI.

## Materials and Methods

### Participants

The study, approved by the institutional review board at Johns Hopkins University School of Medicine, involved signed informed consent from all participants. Thirty-eight persons with chronic cervical or thoracic SCI participated (Table 1), but five datasets were excluded due to excessive motion and one due to missing behavioral measures (see Supplementary Data S1 for detailed quality checking procedures). The final SCI cohort (*n* = 32) had a mean age of 40 years and a Male/Female ratio of 25/7. The ISNCSCI classification included 13 AIS A, 2 AIS B, 11 AIS C, and 6 AIS D participants. A matched healthy control (HC) cohort (mean age = 36 years, M/F ratio = 24/8) was assembled. Twenty HC datasets were acquired as part of this study. Additionally, 12 HC datasets were selected from a previously acquired dataset, specifically the Kirby 21 dataset,^30^ to complete the HC cohort, ensuring it matches the SCI dataset.

**Table 1.** Subject Demographics

	Persons with SCI (*n* = 32)		Healthy controls (*n* = 32; matched)		Statistic value	*p*
	Mean	SD	Mean	SD		
Gender (male/female)	25/7	—	24/8	—	*z* = −1.03	1.69
Age (years)	40.13	13.81	36.53	10.19	*t* = 1.184	0.121
TSI (weeks)	149.63	146.54	—	—	—	—
AIS	A (13), B (2), C (11), D (6)	—	—	—	—	—
Level of Injury	Cervical (16), Thoracic (16)	—	—	—	—	—
Censor Fraction (%)	6.16	7.35	1.21	2.92	*t* = 3.538	<0.001**

AIS, American Spinal Injury Association Impairment Scale; SCI, spinal cord injury; SD, standard deviation; TSI, time since injury.

### Image acquisition

All participants were scanned on a 3T Philips Achieva scanner at the F.M. Kirby Research Center. A T1-weighted MPRAGE structural scan was acquired for each session (6 min, TR/TE/TI = 6.7/3.1/842 ms, resolution = 1 × 1 × 1.2 mm³, SENSE factor = 2, flip angle = 8°). This was followed by two runs of rs-fMRI data using a multislice SENSE-EPI pulse sequence (TR/TE = 2000/30 ms, SENSE factor = 2, flip angle = 75°, 37 axial slices, resolution = 3 × 3 × 3 mm³, 1 mm gap, 32-channel head coil, 200 dynamics). The rs-fMRI runs were always performed after the structural scans to help participants acclimate to the scanner noise and environment. Participants were instructed to stay still with their eyes closed. At the F.M. Kirby Center, routine phantom scans are performed to ensure long-term instrumental reliability (i.e., over weeks and months).

### Brain network construction

**FIG. 1.**
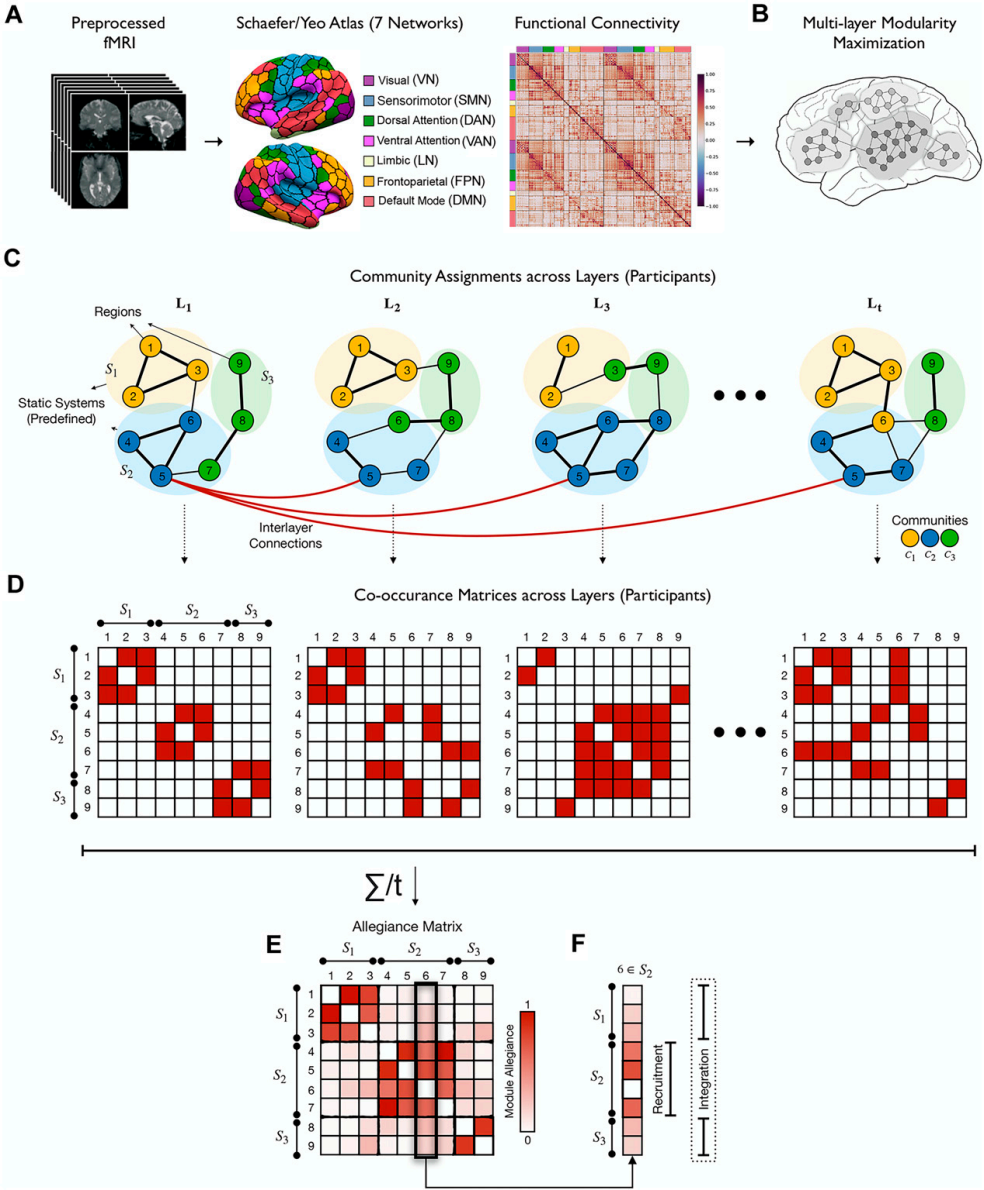
Flowchart illustrating the data analysis process. The flowchart illustrates the utilization of preprocessed rs-fMRI data to compute mesoscale graph measures. **(A)** Functional connectivity matrix is computed by partitioning the preprocessed fMRI data into 200 cortical regions assigned to seven networks. **(B)** Multilayer community detection algorithm is employed to investigate the modular organization of the brain networks. **(C–F)** A toy example illustrates how integration and recruitment coefficients are derived. The example features nine cortical regions (nodes 1–9) and three functional networks (S_1_, S_2_, S_3_) across *t* study participants. **(C)** Multilayer community detection algorithm is used to assign brain regions to three communities (C_1_, C_2_, C_3_) across different study participants (layers defined as study participants: L_1_, L_2_, L_t_). **(D)** Co-occurrence matrices are constructed for each participant, where elements indicate shared community labels between nodes. **(E)** The average of all co-occurrence matrices across participants creates the module allegiance matrix. **(F)** Integration and recruitment coefficients are calculated to measure the consistency of region inclusion in the same functional network and interaction with other networks, respectively. rs-fMRI, resting-state functional MRI.

The flowchart in Figure 1 illustrates the data analysis process. First, we preprocessed anatomical and rs-fMRI data following established protocols, including segmentation, despiking, slice timing correction, motion correction, coregistration, normalization, spatial smoothing, and nuisance regression, as detailed in previous studies^16,31^ and Supplementary Data S1 section. We used preprocessed fMRI data to construct brain networks composed of nodes interconnected by functional edges (Fig. 1A). To specify nodes, we used the Schaefer-Yeo atlas^32^ to partition the brain into 200 cortical regions that are preassigned to one of seven networks: visual (VN), sensorimotor (SMN), dorsal attention (DAN), salience/ventral attention (VAN), limbic (LN), frontoparietal (FPN), and default mode networks (DMN). We then extracted the average Blood Oxygenation Level Dependent time series for each region and calculated the connectivity between each pair of regions using Pearson’s correlation coefficient, converting correlation values to *z*-values with Fisher’s transformation. This resulted in a symmetrical weighted connectivity matrix (adjacency matrix) of 200 × 200 for each participant.

### Multilayer community detection

We employed a multilayer community detection algorithm^21^ to investigate the modular organization of resting-state brain networks in HC and SCI groups (Fig. 1B, C). In this algorithm, each layer represents an individual’s weighted functional connectivity matrix, ensuring consistent community assignments across layers for comparability. “Modular organization” in neuroscience refers to functional or structural groupings. Here, we use it to describe the identification and analysis of functional clusters in resting-state brain networks, applying the multilayer community detection algorithm for quantitative study.

The multilayer modularity function *Q* extends single-slice modularity by considering nodes connected across layers through an interlayer coupling parameter *ω*. The parameters *γ* and *ω* determine module sizes within each layer and the number of modules across layers, respectively. Specifically, the structural resolution *γ* sets the granularity of module identification within each layer, while the interlayer coupling *ω* controls the interaction among modules across layers. The multilayer modularity function is expressed as follows:

(1) $$Q_{\mathrm{multi}-\mathrm{layer}}=\frac{1}{2\mu }\sum_{\mathrm{ijsr}}\left[\left(A_{\mathrm{ijs}}-\gamma V_{\mathrm{ijs}}\right)\delta_{sr}+\omega \delta_{ij}\right]\delta_{\sigma_{is}\sigma_{jr}}$$

where $A_{\mathrm{ijs}}$ and $V_{\mathrm{ijs}}$ are the observed and expected weights of the connection between nodes *i* and *j* in layer *s*, respectively, and μ is the total edge weight in the network. The Kronecker delta function, δ(x,y), is 1 when x=y, and 0 otherwise, assuming that the given network consists of *M* nonoverlapping modules. $\sigma_{is}$ and $\sigma_{jr}$ represent the community assignment of node *i* in slice *s* and node *j* in slice *r*. Choosing appropriate values for *ω* and γ is critical to accurately identifying the modular structure of resting-state brain networks and comparing them across individuals. In this study, we used a 2D parameter space to determine optimal values for *γ* and *ω*. Through this analysis, we found that *γ* = 1.2 and *ω* = 0.1 yielded the highest values of the multilayer modularity function *Q*, indicating the most cohesive and distinguishable modular structures. The community label assignment in the multilayer modularity function *Q* is inherently nondeterministic. To address this, we derived a consensus community assignment by averaging the community assignment values over 50 iterations of the community assignment process.

### Construction of module allegiance matrices

We calculated modularity-related measures using community assignments from the multilayer modularity function (Fig. 1C). From these assignments, we constructed a module allegiance matrix to investigate the community structure. The matrix $P_{ij}$ reflects the fraction of layers where pairs of nodes are assigned to the same community.^26^ To create this matrix, we first built a co-occurrence matrix for each layer/participant (Fig. 1D). The co-occurrence matrix is a square *N-*by*-N* matrix with binary values: 1 if nodes *i* and *j* share a community label, and 0 otherwise. We then averaged these co-occurrence matrices across all layers or participants in each group to form the module allegiance matrix. The elements of this matrix range from 0 to 1, where 0 indicates that nodes *i* and *j* share a community label in no subjects, and 1 indicates they share a label in all subjects (Fig. 1E).

### Computation of recruitment and integration coefficients

We compared community structures between the target groups using two network coefficients from the module allegiance matrices: recruitment and integration coefficients^26^ (Fig. 1F).

The recruitment coefficient measures the fraction of layers in which a node is assigned to the same community as other nodes from the same predefined/static network. The recruitment of node *i* in network *S* is calculated as follows:

(2) $$R_{i}^{S}=\frac{1}{n_{s}}\sum_{j\in S}\;P_{ij}$$

where $n_{s}$ is the number of regions in network *S* and $P_{ij}$ is the module allegiance between node *i* and node *j*.

The integration coefficient measures the fraction of layers in which a given node in network *S* is assigned to the same community as nodes from networks other than *S*. The integration of node *i* in network *S* is calculated as follows:

(3) $$I_{i}^{S}=\frac{1}{N-n_{s}}\sum_{j\notin S}\;P_{ij}$$

where *N* is the total number of brain regions. These coefficients provide a quantitative assessment of the stability and consistency of brain regions’ involvement in functional networks across participants. The recruitment coefficient indicates how consistently a brain region is included in the same functional network, while the integration coefficient shows how consistently a region interacts with other networks. These measures allow assessment of the brain’s modular organization and its relation to behavior and cognition.

### Statistical tests

We employed nonparametric permutation tests with 20,000 repetitions to determine statistically significant group differences between the HC and SCI groups. This involved shuffling the labels 20,000 times to create a null distribution, that is, calculating the allegiance matrix difference between groups in each shuffled dataset and comparing it with the actual group difference. The advantage of this approach is that it does not rely on distributional assumptions.^33^ To control for multiple comparisons, we applied the Benjamini and Hochberg false discovery rate (FDR) correction^34^ to all statistical tests, with a significance threshold (q) of 0.05.

## Results

Table 1 provides detailed demographic and clinical data for the study participants. There were no significant differences between HC and SCI cohorts in gender distribution (HC: 25 males, 7 females; SCI: 24 males, 8 females) or age (HC: 40.1 ± 13.8 years; SCI: 36.5 ± 10.2 years; *p* > 0.1). However, a significant difference was observed in censor fraction due to motion and imaging artifacts (HC: 1.21% vs. SCI: 6.16%; *p* < 0.001). The average time since injury for SCI participants was 150 ± 147 weeks, and SCI severity was assessed using the AIS.

**FIG. 2.**
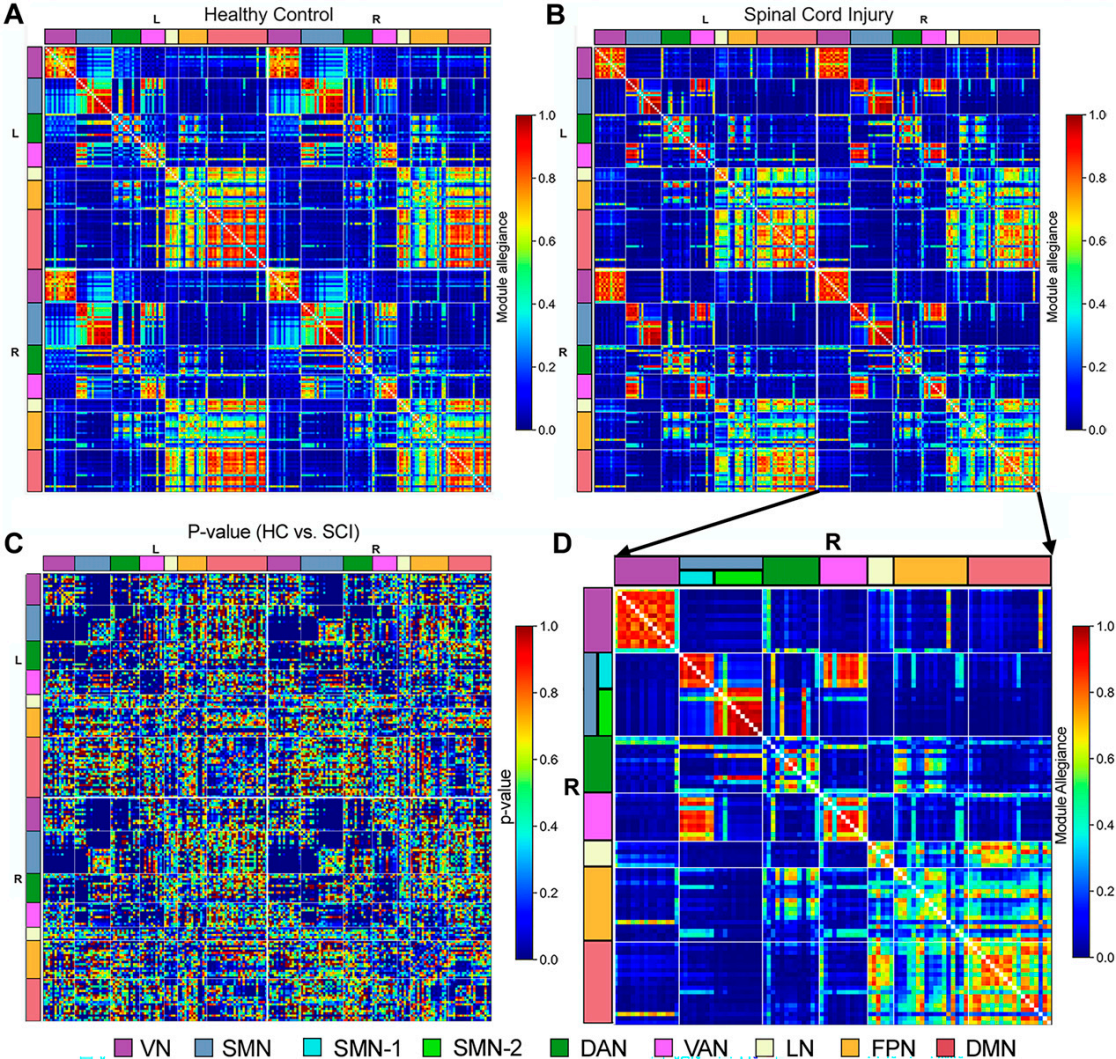
Group differences in module allegiance between healthy controls (HC) and spinal cord injury (SCI) cohorts. Panels illustrate **(A)** HC cohort module allegiance matrix, **(B)** SCI cohort module allegiance matrix, **(C)** significant *p* values for cohort differences, and **(D)** detailed view focusing on right-hemisphere nodes in the SCI cohort. The SCI cohort’s module allegiance matrix reveals that the parcels within the sensorimotor network (SMN) segregate into two clusters, marked by decreased module allegiance values between the clusters. These clusters are color-coded and labeled as SMN-1 (cyan) and SMN-2 (lime). L, left hemisphere; R, right hemisphere.

Module allegiances reveal the consistency of community structure across individuals (Fig. 2A, B). Figure 2C presents a *p* value matrix indicating significant group differences in module allegiance values between HC and SCI cohorts (*p* < 0.001; adjusted for FDR). Significant differences are observed across all seven major networks, with more noticeable changes in the SMN, DAN, VAN, and DMN networks. Figure 2D provides a detailed view of the right-hemisphere nodes in the SCI cohort, magnifying the fourth quadrant of the module allegiance matrix from Figure 2B. This enhanced view highlights distinct clusters within the SMN. Specifically, parcels within the SMN are segregated into two clusters with decreased module allegiance values between them. This segregation is clearly marked in Figure 2D, with the border between these clusters denoted by color-coded labels: SMN-1 (cyan) and SMN-2 (lime).

**FIG. 3.**
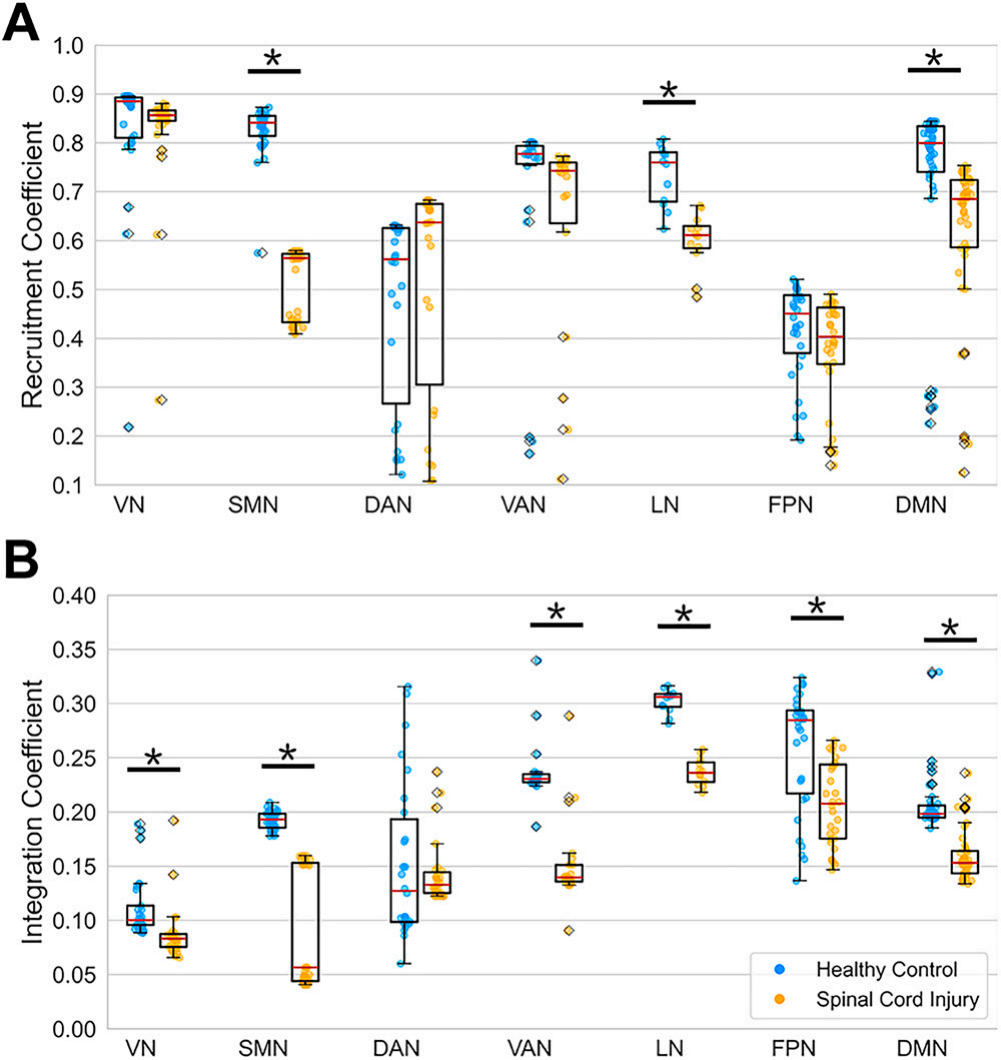
Recruitment and integration coefficients across networks in healthy controls (HC) and spinal cord injury (SCI) cohorts. **(A)** Box plot of recruitment coefficients in HC and SCI cohorts, with asterisks denoting statistically significant differences (*p* < 0.001, FDR corrected) for some networks. **(B)** Box plot of integration coefficients in HC and SCI cohorts, similarly annotated. Networks represented include visual (VN), sensorimotor (SMN), dorsal attention (DAN), salience/ventral attention (VAN), limbic (LN), frontoparietal (FPN), and default mode (DMN) networks. FDR, false discovery rate.

Figure 3A and B provides additional insight by displaying the distribution of recruitment and integration coefficient values for each functional network using box plots. Each dot in these plots represents individual cortical regions from the Schaefer-Yeo Atlas assigned to each functional network, averaged across each hemisphere. In Figure 3A, reduced recruitment coefficients are observed within the SMN, LN, and DMN in the SCI cohort compared to HC. Figure 3B complements this observation by revealing decreased integration coefficients in the VN, SMN, VAN, LN, FPN, and DMN among SCI participants relative to HC. These findings underscore altered network integration across multiple brain networks in persons with SCI.

**FIG. 4.**
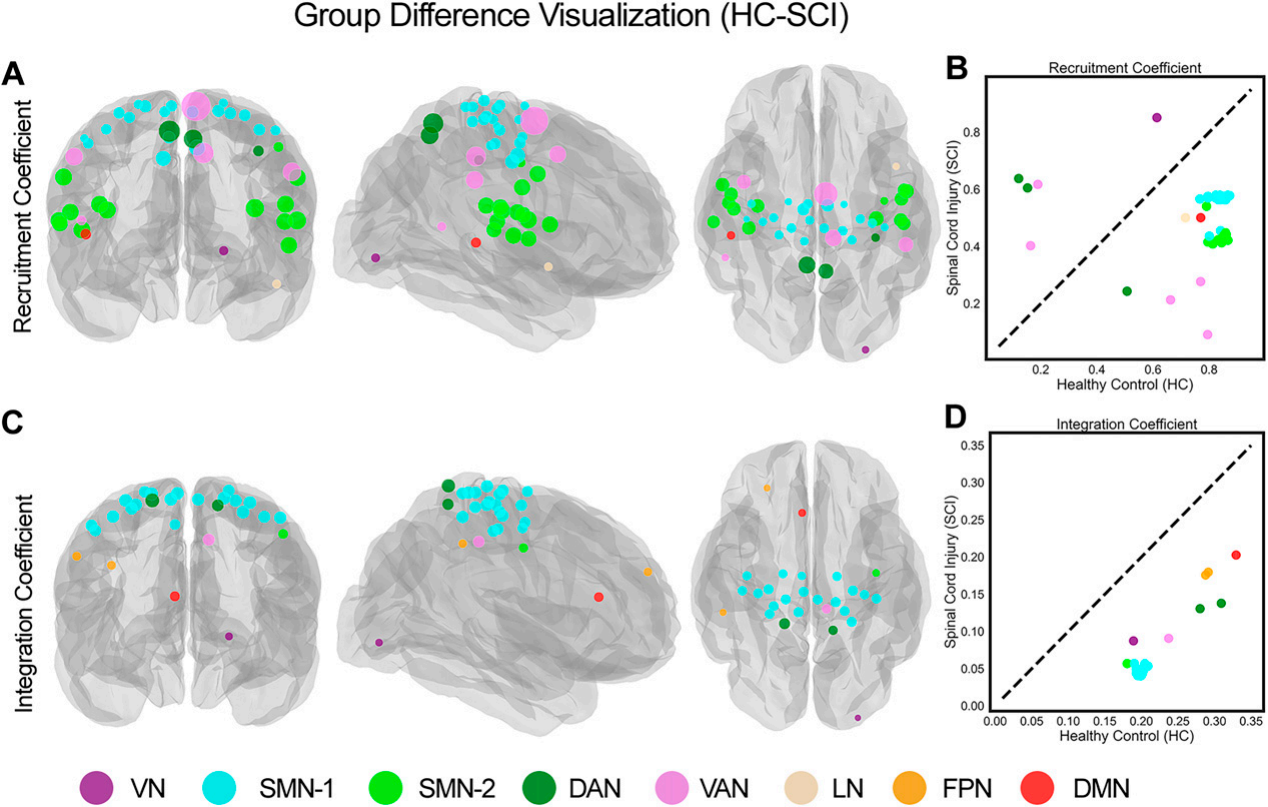
Visualization of parcels with significant group differences in recruitment and integration coefficients between healthy controls (HC) and spinal cord injury (SCI) cohorts **(A)** Visualization of parcels with significantly different recruitment coefficients in the SCI cohort compared to the HC cohort (HC-SCI; *p* > 0.001, FDR corrected). Circle sizes represent the magnitude of differences. **(B)** Scatter plot comparing parcels based on SCI and HC recruitment coefficients. The diagonal dotted line indicates the identity line. Parcels below this line show significantly lower recruitment coefficients in the SCI cohort compared to the HC cohort. **(C)** Visualization of parcels with significant group differences in integration coefficients between the HC and SCI cohorts (HC-SCI; *p* > 0.001, FDR corrected). **(D)** Scatter plot comparing parcels based on SCI and HC integration coefficients. Networks represented include visual (VN), sensorimotor (SMN), dorsal attention (DAN), salience/ventral attention (VAN), limbic (LN), frontoparietal (FPN), and default mode (DMN) networks. FDR, false discovery rate.

Figure 4A and C employed glass-brain visualizations to display the locations of the parcels that exhibit significant differences (*p* > 0.001, FDR corrected) in the recruitment and integration coefficients between the HC and SCI cohorts. The visualizations show transparent views of both hemispheres with overlapping projections in coronal, sagittal, and axial views and highlight a distinct geographical separation between SMN-1 and SMN-2 regions. Specifically, SMN-1 parcels were found in the dorsolateral and paramedian regions, commonly linked to upper and lower body functions, while SMN-2 parcels were located in the ventrolateral region, typically associated with orofacial functions. Furthermore, Figure 4B shows that SMN-1 and SMN-2 parcels are tightly clustered with similar recruitment coefficient values, although SMN-2 parcels exhibit a more noticeable decline in these values compared to SMN-1 parcels. Note that Figure 4B and D shows scatter plots that can be used to compare parcels based on their recruitment and integration coefficients. A diagonal dotted line in these plots acts as a reference, indicating the direction of group differences: parcels below the line have significantly lower recruitment coefficients in the SCI cohort compared to the HC cohort, while those above the line have higher recruitment coefficients. Figure 4D also reveals a consistent pattern among parcels showing significant differences in integration coefficients: the SCI cohort consistently exhibits lower integration coefficients across these parcels. The situation with recruitment coefficients is more nuanced, as shown in Figure 4B. Beyond the SMN parcels, numerous parcels within the same functional network, like the VAN, exhibit varied changes between the SCI and HC cohorts, with some increasing and others decreasing. Interestingly, the functional segregation between SMN-1 and SMN-2 parcels was more pronounced in persons with thoracic SCI than in those with cervical injuries, as detailed in the Supplementary Text and illustrated in Supplementary Figures S2 and Figure S3.

## Discussion

Our study used a multilayer community detection approach to examine brain network alterations in chronic SCI. We found significant differences in community structures between the SCI and HC cohorts, with altered module allegiance and distinct clustering patterns, especially within the SMN, highlighting SCI’s impact on brain connectivity.

The significant differences in module allegiance values between the cohorts (Fig. 2) underscore the profound impact of SCI on brain community structures. Reduced recruitment coefficients within the SMN, LN, and DMN (Fig. 3A) suggest a diminished ability of these networks to maintain cohesive community structures in persons with SCI, aligning with previous research indicating disrupted functional connectivity in these regions.^14,35,36^ Furthermore, decreased integration coefficients across multiple brain networks, including the VN, SMN, VAN, LN, FPN, and DMN, in the SCI cohort (Fig. 3B) highlight widespread disruptions in network integration. These reductions suggest that persons with SCI experience impaired internetwork communication, contributing to the sensory and motor deficits commonly observed in this population.

Significantly, we observed a notable segregation within the SMN, where parcels were divided into two distinct clusters (SMN-1 and SMN-2; Figs. 2 and 4). The anatomical separation of these clusters, with SMN-1 associated with upper and lower body functions and SMN-2 linked to orofacial functions (Fig. 4A), offers a new perspective on the functional reorganization of the SMN cortex following SCI. This segregation was more pronounced in persons with thoracic injuries, suggesting that the level of spinal injury may influence the degree of functional reorganization within the brain (Supplementary Figs. S3 and Figs. S4). The consistent pattern of lower integration coefficients across parcels in the SCI cohort (Fig. 4D) indicates a pervasive reduction in network integration. However, the recruitment coefficients exhibited more variability, with some parcels showing increased values in SCI participants. This variability, such as within the VAN, suggests that the impact of SCI on network recruitment is complex and may involve compensatory mechanisms (Fig. 4B).

These observed patterns of segregation and variability in network recruitment and integration are likely linked to underlying disruptions in motor and sensory pathways caused by SCI. SCI leads to neuroinflammation, apoptosis, and synaptic plasticity changes.^37–39^ These alterations, affecting both the spinal cord and cortex, likely contribute to the cortical reorganization seen in our study, particularly within the SMN, with subcortical regions such as the thalamus and basal ganglia playing a critical role in modulating these effects.

Such alterations in brain network dynamics may have clinical implications. Reduced recruitment and integration within key functional networks may underlie the sensory and motor impairments experienced by persons with SCI. Understanding these network-level changes may inform the development of targeted rehabilitation strategies aimed at enhancing brain network integration and improving functional outcomes in this population.

The multilayer community detection algorithm used in this study effectively captures both individual variability and group-level brain network patterns by treating each subject as a separate layer. This is particularly useful in heterogeneous populations like SCI. However, the method is computationally demanding and requires careful tuning of parameters, such as interlayer coupling. Additionally, it assumes direct comparability of functional networks across individuals, which may overlook individual differences. Despite these challenges, this approach offers a powerful tool for identifying subtle network alterations that single-layer methods may miss.

While our study provides valuable insights into brain network alterations in chronic SCI, several limitations remain. The cross-sectional design limits causal inference, and longitudinal studies are needed to examine temporal dynamics and functional recovery. Our sample size, though adequate for group differences, may limit the generalizability of our findings and hinder more detailed classification based on injury severity and level. The cohort’s heterogeneity, particularly in terms of severity and time since injury, further complicates interpretation. A larger sample size would help address potential confounding factors such as age, sex, pre-existing conditions, and injury duration. Replicating the study with a larger cohort could validate our results and clarify whether the observed brain network differences are primarily due to spinal cord damage or other contributing factors, thereby providing a more robust foundation for future research and clinical interventions.

## Conclusion

This study used a multilayer community detection approach to examine brain network changes in individuals with chronic SCI. Significant alterations were found in community structures, particularly within the SMN, showing segregation aligned with body region functions. Reduced recruitment and integration coefficients across multiple networks suggest impaired internetwork communication, potentially contributing to sensory and motor challenges. These insights are key to informing targeted rehabilitation strategies.

## Transparency, Rigor, and Reproducibility Statement

This study involves a post hoc analysis of datasets. As a result, the study’s registration, analytic plans, and calculations for statistical power and sample size were not conducted prior to data acquisition. The study employs a subset of datasets from another study, which was registered after it had started; the registration information can be found at https://clinicaltrials.gov/study/NCT03854214. Additionally, the study includes datasets from studies that were not formally registered because they were noninterventional and did not meet the criteria for mandatory registration on clinical trial registries. The analysis plan was not preregistered officially. In total, 92 potential participants underwent screening, and imaging data were obtained from 38 participants and successfully analyzed in 32. Imaging acquisition and analyses were carried out by team members who were blinded to relevant participant characteristics, and clinical outcomes were assessed by team members who were blinded to imaging results. All equipment and software used for imaging and preprocessing are commercially available. The key inclusion criteria and outcome evaluations follow established standards. The test–retest reliability of the primary clinical outcome measure has not been formally determined. De-identified data from this study are not publicly archived but may be assessed for potential sharing in accordance with institutional review board standards by contacting the corresponding author. Likewise, the analytic code used for the study’s analyses is not found in a public repository but may be evaluated for possible sharing by emailing the corresponding author. The availability of both the data and analytic code is contingent upon a situation-specific assessment.

## Abbreviations Used

AIS: ASIA Impairment Scale
ASIA: American Spinal Injury Association
DAN: Dorsal attention network
DMN: Default mode networks
FDR: False discovery rate
FPN: Frontoparietal network
HC: Healthy control
ISNCSCI: International Standards for Neurological Classification of Spinal Cord Injury
LN: Limbic network
MPRAGE: Magnetization Prepared Rapid Acquisition Gradient Echo
rs-fMRI: Resting-state functional MRI
SCI: Spinal cord injury
SENSE-EPI: Sensitivity Encoding Echo Planar Imaging
SMN: Sensorimotor network
TR/TE: Repetition Time / Echo Time
TSI: Time Since Injury
VAN: Salience/ventral attention network
VN: Visual network
