# Supplementary material for: Brain Network Alterations in Chronic Spinal Cord Injury: Multilayer Community Detection Approach
Source: Neurotrauma Rep. 2024 Nov 6;5(1):1048–59. doi: 10.1089/neur.2024.0098 (PMC11685503; doi:10.1089/neur.2024.0098)
Supplement: Supplementary Figure S1 [file neur.2024.0098_supp_figs1.docx]

## Supplementary Figures


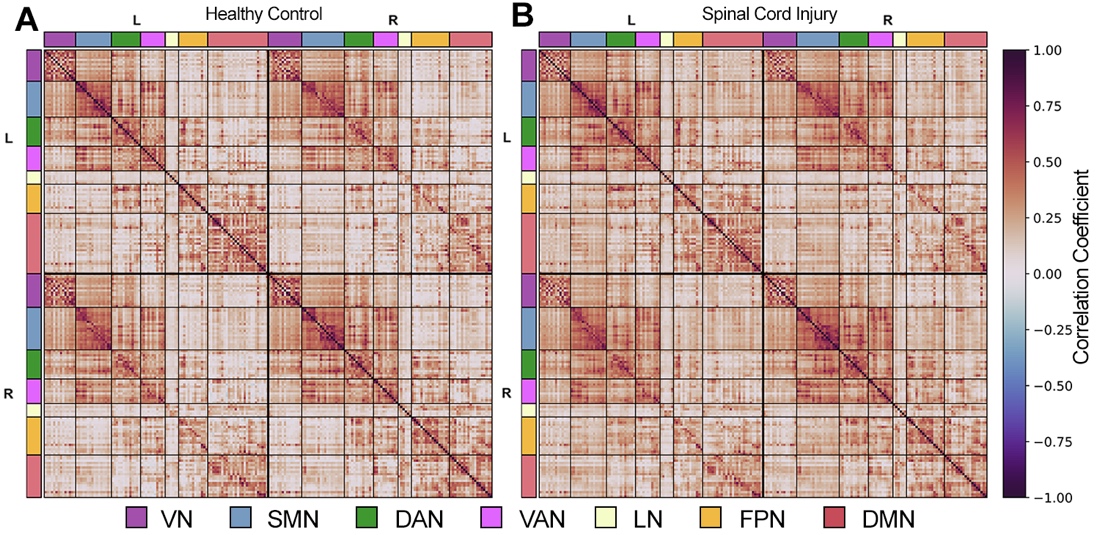


Figure S1. Functional connectivity matrices, averaged across subjects, are shown for healthy controls and individuals with spinal cord injury. (A) shows the matrix for healthy controls (HC), and (B) shows the matrix for individuals with spinal cord injury (SCI). These matrices depict the correlation between preprocessed rsfMRI signal time courses of various cortical regions. Color represents the strength of these correlations. The networks represented in these matrices include Visual (VN), Sensorimotor (SMN), Dorsal Attention (DAN), Salience/Ventral Attention (VAN), Limbic (LN), Frontoparietal (FPN), and Default Mode (DMN) networks. (L: left hemisphere; R: right hemisphere).
