# Supplementary material for: Brain Network Alterations in Chronic Spinal Cord Injury: Multilayer Community Detection Approach
Source: Neurotrauma Rep. 2024 Nov 6;5(1):1048–59. doi: 10.1089/neur.2024.0098 (PMC11685503; doi:10.1089/neur.2024.0098)
Supplement: Supplementary Figure S2 [file neur.2024.0098_supp_figs2.docx]

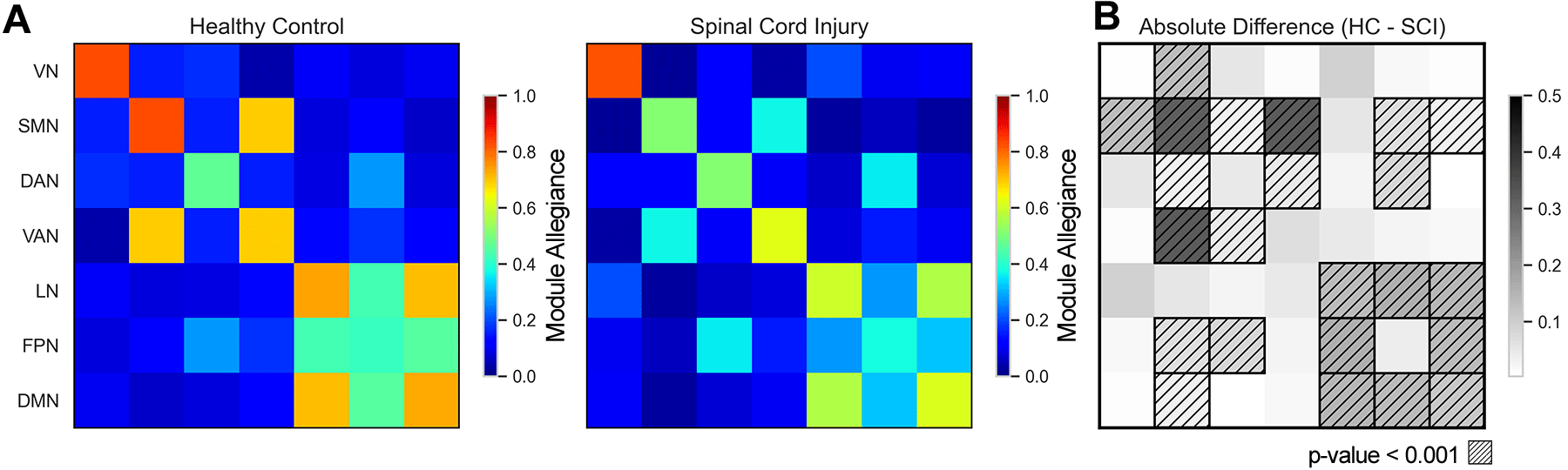
Figure S2. Network-level module (coarse) allegiance matrices and graph measure profiles. (A) Network-level module (coarse) allegiance matrices are shown for both the healthy control (HC) and spinal cord injury (SCI) cohorts. (B) Absolute significant differences between the two cohorts are visualized.
