# Supplementary material for: Brain Network Alterations in Chronic Spinal Cord Injury: Multilayer Community Detection Approach
Source: Neurotrauma Rep. 2024 Nov 6;5(1):1048–59. doi: 10.1089/neur.2024.0098 (PMC11685503; doi:10.1089/neur.2024.0098)
Supplement: Supplementary Figure S3 [file neur.2024.0098_supp_figs3.docx]

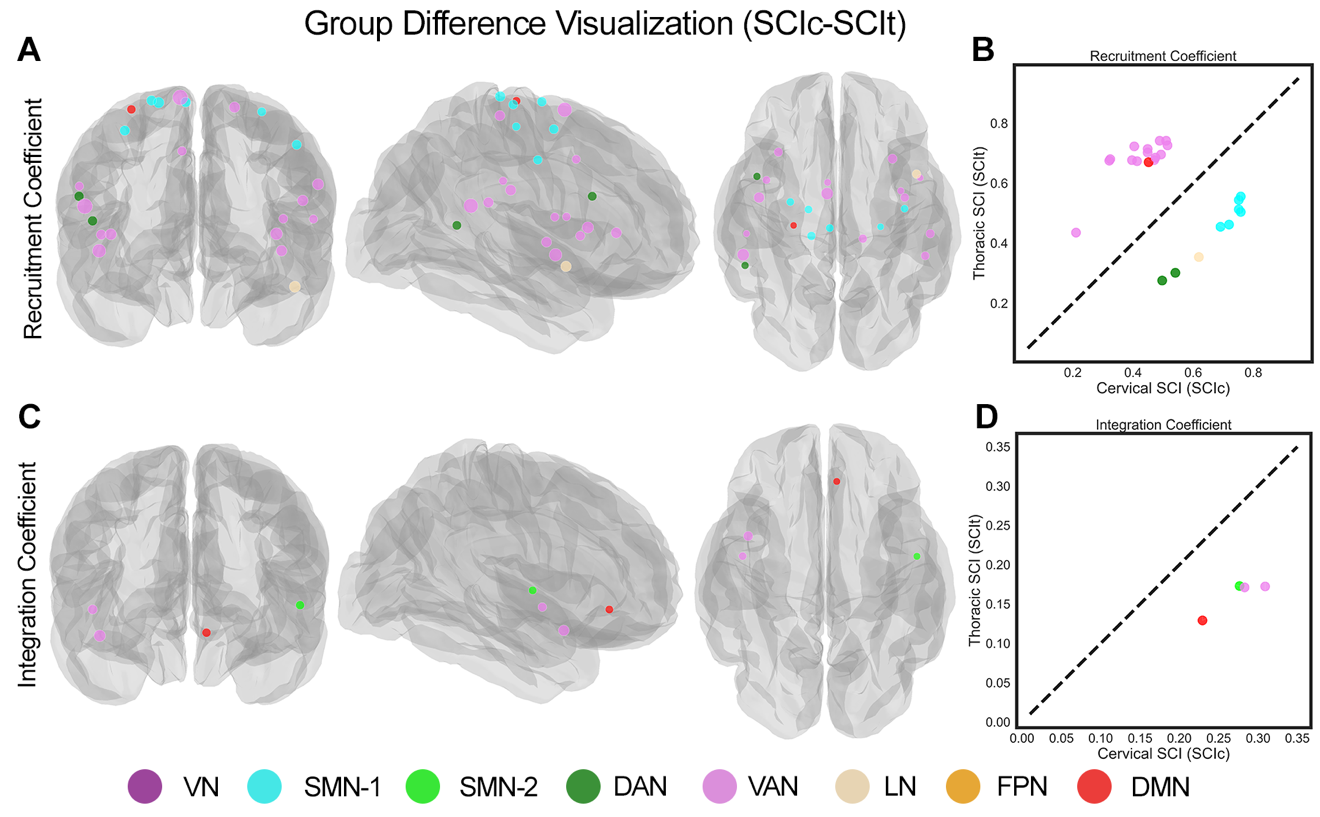


Figure S3. Group differences in recruitment and integration coefficients between individuals with cervical spinal cord injury (SCIc) and thoracic spinal cord injury (SCIt). (A) Parcels with significantly different recruitment coefficients in the SCIt cohort compared to SCIc (p > 0.001, FDR corrected). Circle sizes indicate the magnitude of differences. (B) Scatter plot of recruitment coefficients, with parcels below the diagonal line showing lower values in SCIt. (C) Parcels with significant differences in integration coefficients between SCIc and SCIt (p > 0.001, FDR corrected). (D) Scatter plot of integration coefficients. Networks are: Visual (VN), Sensorimotor (SMN), Dorsal Attention (DAN), Salience/Ventral Attention (VAN), Limbic (LN), Frontoparietal (FPN), and Default Mode (DMN).
