# Supplementary material for: Brain Network Alterations in Chronic Spinal Cord Injury: Multilayer Community Detection Approach
Source: Neurotrauma Rep. 2024 Nov 6;5(1):1048–59. doi: 10.1089/neur.2024.0098 (PMC11685503; doi:10.1089/neur.2024.0098)
Supplement: Supplementary Figure S4 [file neur.2024.0098_supp_figs4.docx]

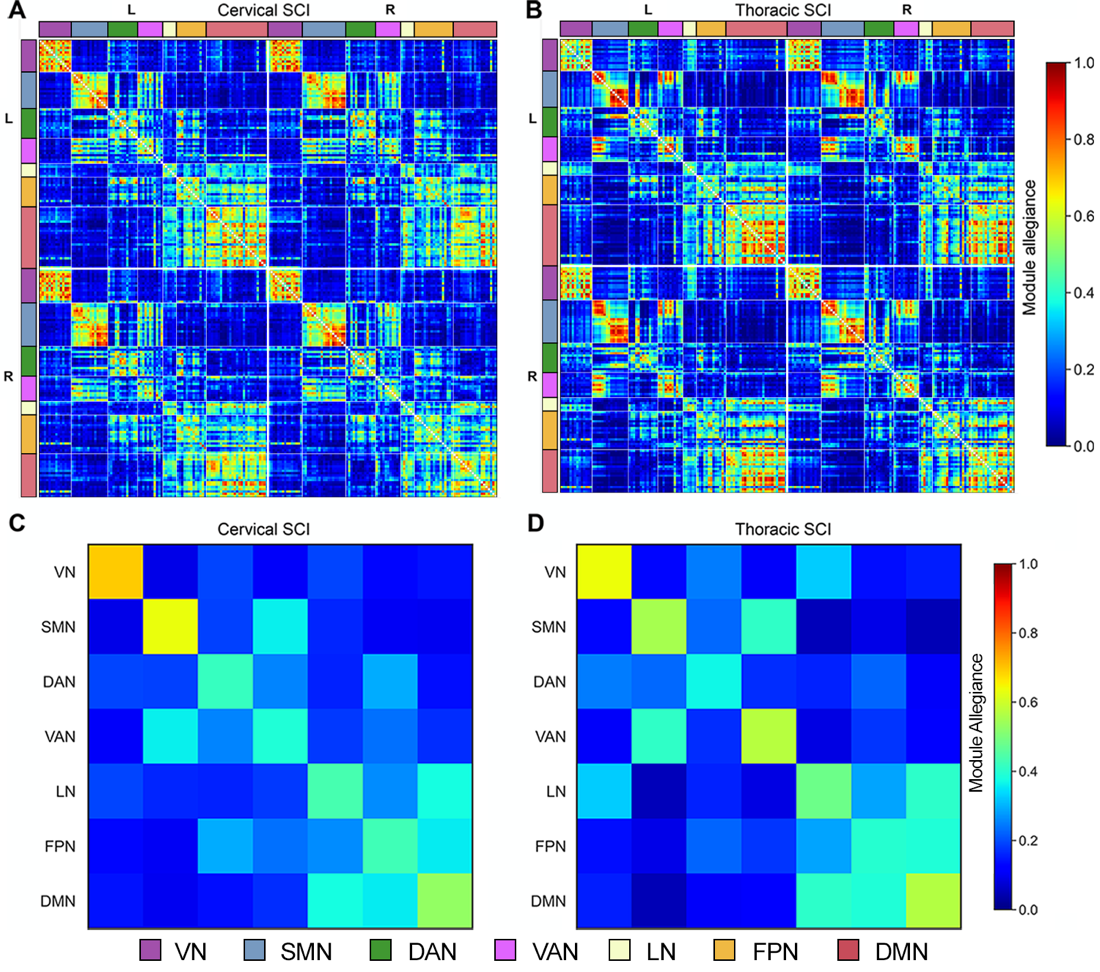


Figure S4. Module allegiance matrices for cervical and thoracic spinal cord injury (SCI) cohorts. (A) Parcel-level matrices for cervical SCI. (B) Parcel-level matrices for thoracic SCI. (C) Network-level matrices for cervical SCI. (D) Network-level matrices for thoracic SCI. (L: left hemisphere; R: right hemisphere). Networks are: Visual (VN), Sensorimotor (SMN), Dorsal Attention (DAN), Salience/Ventral Attention (VAN), Limbic (LN), Frontoparietal (FPN), and Default Mode (DMN).
