# Supplementary material for: Brain Network Alterations in Chronic Spinal Cord Injury: Multilayer Community Detection Approach
Source: Neurotrauma Rep. 2024 Nov 6;5(1):1048–59. doi: 10.1089/neur.2024.0098 (PMC11685503; doi:10.1089/neur.2024.0098)
Supplement: Supplementary Data S1 [file neur.2024.0098_supp_datas1.docx]

# Supplementary Text

## Supplementary Methods

### Study participants

The inclusion and exclusion criteria for study participants are as follows. Healthy participants were matched to the injured group based on demographic characteristics such as age, sex, and ethnicity.

Inclusion Criteria:

- Males and females aged 18-99 from all ethnic backgrounds.
- Traumatic SCI at the cervical or thoracic levels without lower motor neuron involvement.
- ASIA grades A-D, chronic injury >6 months post-injury.
- Participants are in good health generally, with the ability to comply with study protocols.

Exclusion Criteria:

- Contraindications to MRI (e.g., pacemakers, claustrophobia).
- History of moderate/severe brain injury or major spine deformities.
- Severe spasticity or movement disorders preventing imaging.
- Pregnancy, concurrent lower motor neuron disease, or unstable long bone fractures.
- Intolerance to electrical stimulation or recent COVID-19 diagnosis (past 28 days).

### Preprocessing

The study utilized a series of image-processing steps to analyze both anatomical and fMRI data. To perform whole brain segmentation of the anatomical images, an automated pipeline provided by FreeSurfer^1^ was utilized, which involved identifying and labeling different brain regions based on their anatomical features. After whole brain segmentation, the rsfMRI data was preprocessed using the Analysis of Functional NeuroImages (AFNI) software^2^. The preprocessing pipeline followed the recommendations outlined by Jo et al.^3^ and included de-spiking, slice timing correction, motion correction, co-registration, normalization, spatial smoothing, and nuisance regression. Specifically, de-spiking involved identifying spikes in the rsfMRI data based on intensity deviation from a smooth L1 fit to a voxel’s time series relative to the time series variance. Slice timing correction corrected for differences in the time at which different slices of the rsfMRI data were acquired. Functional and anatomical data were aligned, followed by co-registration of the functional volumes to a functional volume with minimum outlier fraction. The outcome of this procedure was then utilized to assess the functional data for the presence of head motion artifacts. The sudden motion detection feature of the AFNI was implemented, employing a threshold level of 0.4 mm for the Euclidean L2 norm of motion displacement during each repetition time (TR) interval. Normalization of anatomical data involved the use of non-linear registration to a Montreal Neurological Institute (MNI) template. During spatial smoothing, a 6 mm full-width at half-maximum Gaussian kernel was applied to smooth the rsfMRI data, reducing noise and improving the signal-to-noise ratio. In the Nuisance regression step, censoring of motion TRs, nuisance regression, and bandpass filtering were conducted simultaneously in a single regression model. Specifically, motion parameters and derivatives from each run, the signal from the first three principal components of lateral ventricles, and the signal from locally averaged white matter^4^ were regressed out.

### Construction of Network-level Module Allegiance Matrices

To better summarize information in the parcel-level module allegiance matrix and enhance our comprehension of the data, we expanded our investigation from a parcel to a network level. This entailed developing a brain network-level module allegiance matrix. To accomplish this, we took the original parcel-level module allegiance matrix and averaged values from all parcel pairings across all functional networks. Following this, we further averaged the data across hemispheres, producing a compact 7x7 network-level module allegiance matrix. We then computed and compared the recruitment and integration coefficients between the HC and SCI cohorts at the brain network level. The resulting network-level module allegiance matrix is shown in Figure S2.

### Investigation of SCI sub-cohorts: individuals with cervical and thoracic SCI

We also calculated the parcel- and network-level module allegiance matrices for subgroups within the SCI cohort, as shown in Figure S4. Specifically, we divided the SCI cohort into two subgroups: those with cervical vs. thoracic injuries. This division allowed us to qualitatively compare the resulting module allegiance matrices with those obtained from the entire SCI cohort. Intriguingly, this functional segregation of SMN-1 and SMN-2 parcels appears more pronounced in individuals with thoracic spinal cord injuries than in those with cervical injuries, as shown in Figures S3 and S4.

## Supplementary Discussion

### Neural Pathway Disruptions and Subcortical Contributions to Cortical Reorganization

Preclinical studies have shown that spinal cord injury (SCI) disrupts key motor and sensory pathways, including the corticospinal and spinothalamic tracts, leading to significant functional impairments ^5–7^. After SCI, processes such as neuroinflammation, apoptosis, and changes in synaptic plasticity hinder recovery and contribute to neural reorganization at both the spinal and cortical levels. Neuroinflammation exacerbates tissue damage and impairs neural signaling, while apoptosis in neurons and glial cells further disrupts signal transmission along these pathways ^8,9^. These alterations in synaptic plasticity, affecting both the spinal cord and cortex, likely play a role in the cortical reorganization observed in our study, particularly within the sensorimotor network.

Subcortical regions, particularly the thalamus, basal ganglia, and brainstem, are also integral to this reorganization. These regions act as relay centers that modulate sensory and motor information between the spinal cord and cortex. The thalamus, for example, processes inputs from ascending sensory pathways such as the dorsal column-medial lemniscus (DCML) and spinothalamic tracts, which carry touch, proprioception, and pain signals ^10^. Disruptions to these pathways after SCI affect sensory processing, which in turn influences cortical reorganization. Similarly, descending motor pathways, particularly the corticospinal tract (CST), are often impaired, leading to motor dysfunction and compensatory changes in cortical motor areas ^11,12^. Compensatory mechanisms may activate alternative pathways, such as the rubrospinal and reticulospinal tracts, contributing to reorganization in both cortical and subcortical structures ^13^.

The involvement of subcortical regions such as the basal ganglia and brainstem is critical for modulating motor output and adapting to changes in sensory input. These structures undergo significant connectivity changes after SCI, further contributing to cortical reorganization, particularly within the sensorimotor networks ^10,14^. This intricate interplay between disrupted spinal pathways and subcortical relays highlights the complex, multi-level nature of cortical reorganization following SCI.

# References

1. Reuter M, Schmansky NJ, Rosas HD, et al. Within-subject template estimation for unbiased longitudinal image analysis. NeuroImage 2012;61(4):1402–1418; doi: 10.1016/j.neuroimage.2012.02.084.

2. Cox RW. AFNI: software for analysis and visualization of functional magnetic resonance neuroimages. Comput Biomed Res Int J 1996;29(3):162–173; doi: 10.1006/cbmr.1996.0014.

3. Jo HJ, Gotts SJ, Reynolds RC, et al. Effective Preprocessing Procedures Virtually Eliminate Distance-Dependent Motion Artifacts in Resting State FMRI. J Appl Math 2013;2013; doi: 10.1155/2013/935154.

4. Jo HJ, Reynolds RC, Gotts SJ, et al. Fast detection and reduction of local transient artifacts in resting-state fMRI. Comput Biol Med 2020;120:103742; doi: 10.1016/j.compbiomed.2020.103742.

5. Borrell JA, Krizsan-Agbas D, Nudo RJ, et al. Effects of a contusive spinal cord injury on cortically-evoked spinal spiking activity in rats. J Neural Eng 2020;17(6):066005; doi: 10.1088/1741-2552/abc1b5.

6. Friedli L, Rosenzweig ES, Barraud Q, et al. Pronounced species divergence in corticospinal tract reorganization and functional recovery after lateralized spinal cord injury favors primates. Sci Transl Med 2015;7(302):302ra134-302ra134; doi: 10.1126/scitranslmed.aac5811.

7. Oudega M, Perez MA. Corticospinal reorganization after spinal cord injury. J Physiol 2012;590(16):3647–3663; doi: 10.1113/jphysiol.2012.233189.

8. Abbaszadeh F, Fakhri S, Khan H. Targeting apoptosis and autophagy following spinal cord injury: Therapeutic approaches to polyphenols and candidate phytochemicals. Pharmacol Res 2020;160:105069; doi: 10.1016/j.phrs.2020.105069.

9. Beattie MS. Inflammation and apoptosis: linked therapeutic targets in spinal cord injury. Trends Mol Med 2004;10(12):580–583; doi: 10.1016/j.molmed.2004.10.006.

10. Jutzeler CR, Huber E, Callaghan MF, et al. Association of pain and CNS structural changes after spinal cord injury. Sci Rep 2016;6(1):18534; doi: 10.1038/srep18534.

11. Cronin AE, Detombe SA, Duggal CA, et al. Spinal cord compression is associated with brain plasticity in degenerative cervical myelopathy. Brain Commun 2021;3(3):fcab131; doi: 10.1093/braincomms/fcab131.

12. Mohammed H, Hollis ER. Cortical Reorganization of Sensorimotor Systems and the Role of Intracortical Circuits After Spinal Cord Injury. Neurotherapeutics 2018;15(3):588–603; doi: 10.1007/s13311-018-0638-z.

13. Filli L, Schwab ME. The rocky road to translation in spinal cord repair. Ann Neurol 2012;72(4):491–501; doi: 10.1002/ana.23630.

14. Karunakaran KD, Yuan R, He J, et al. Resting-State Functional Connectivity of the Thalamus in Complete Spinal Cord Injury. Neurorehabil Neural Repair 2020;34(2):122–133; doi: 10.1177/1545968319893299.
